# Direct oral anticoagulants do not affect miR-27a-3p expression, a regulator of coagulation cascade, in atrial fibrillation patients

**DOI:** 10.1007/s11239-025-03102-5

**Published:** 2025-04-21

**Authors:** Georgia Ragia, Myria Pallikarou, Chrysoula Michou, Thomas Thomopoulos, Georgios Chalikias, Athanasios Trikas, Dimitrios N. Tziakas, Vangelis G. Manolopoulos

**Affiliations:** 1 Laboratory of Pharmacology, Medical School, Democritus University of Thrace, Dragana Campus, Alexandroupolis, 68100 Greece; 2 Individualised Medicine & Pharmacological Research Solutions (IMPReS) Center, Dragana Campus, Alexandroupolis, 68100 Greece; 3 Department of Cardiology, “Elpis” General Hospital of Athens, Athens, Greece; 4 Cardiology Department, Medical School, Democritus University of Thrace, Dragana Campus, Alexandroupolis, 68100 Greece; 5 Department of Cardiology, Evaggelismos Hospital, Athens, Greece; 6 Clinical Pharmacology Unit, Academic General Hospital of Alexandroupolis, Dragana Campus, Alexandroupolis, 68100 Greece; 7 Laboratory of Pharmacology, School of Pharmacy, Aristotle University of Thessaloniki, Thessaloniki, 54124 Greece

**Keywords:** MicroRNAs, miR-27a, Direct oral anticoagulants, Rivaroxaban, Apixaban, Dabigatran

## Abstract

miR-27a-3p targets several proteins on the coagulation cascade. The potential effect of direct oral anticoagulants (DOACs) treatment on miR-27a-3p expression and their broader regulative effect on anticoagulation is unknown. Fifty-nine atrial fibrillation patients treated with rivaroxaban (*n* = 19), apixaban (*n* = 27) or dabigatran (*n* = 13), were included in the study. miR-27a-3p expression was analyzed at baseline and after 7 days of DOAC therapy by using a predesigned TaqMan assay. Relative quantitation of miR-27a-3p expression was calculated and compared in pooled population and in different sample groups. DOAC therapy did not alter miR-27a-3p expression (0.80 fold-change, *p* = 0.486, pooled population; 0.839 fold-change, *p* = 0.706, rivaroxaban; 0.921 fold-change, *p* = 0.800, apixaban; 0.733 fold-change, *p* = 0.540, dabigatran). miR-27a-3p expression did not differ between controls and bleeding cases (0.833 fold-change, *p* = 0.588, baseline). Female patients had a trend towards increased baseline expression (1.564 fold-change, *p* = 0.177) and reduced expression after DOAC treatment (0.683 fold-change, *p* = 0.243) compared to male patients. Despite the regulatory role of miR-27a-3p on coagulation cascade, treatment with DOACs did not alter its expression. However, additional studies in different ethnic groups are necessary to fully elucidate the effect, if any, of DOACs on miR-27a-3p expression.

**Graphical Abstract:**

**Figure:**
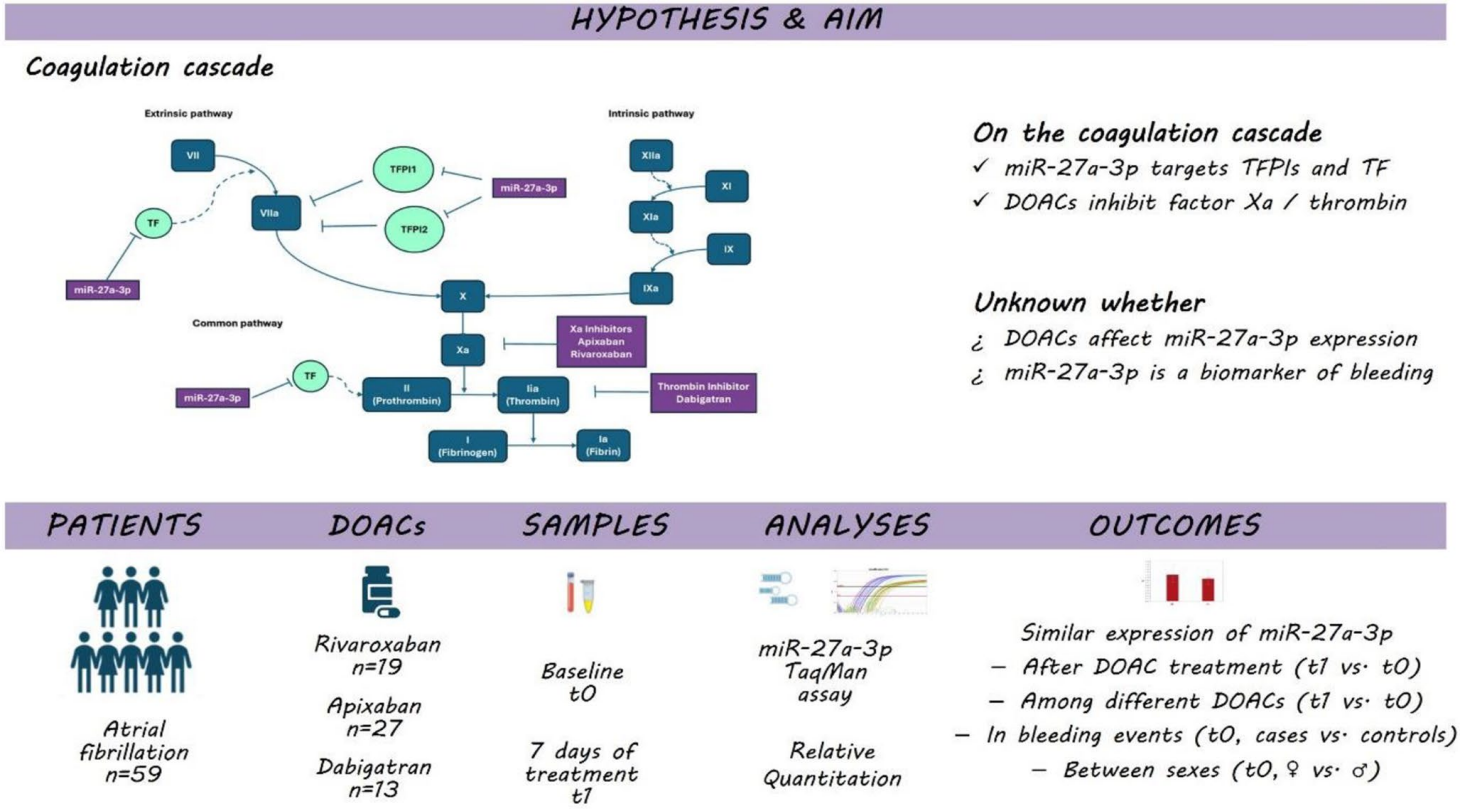
Direct oral anticoagulant effect on miR-27a-3p expression in atrial fibrillation. Both miR-27a-3p and direct oral anticoagulants (DOACs) act on the coagulation cascade. Following over time changes in miR-27a-3p expression in DOAC treated atrial fibrillation patients, we have shown that DOAC treatment does not alter its expression

## Keypoints

• miR-27a-3p targets tissue factor pathway inhibitor, tissue factor pathway inhibitor 2 and tissue factor on the coagulation cascade.
• Whether treatment with DOACs alters the expression of miR-27a-3p leading to a broader regulation of the coagulation cascade is currently unknown.
• In 59 atrial fibrillation (AF) patients treated with rivaroxaban (*n* = 19), apixaban (*n* = 27) or dabigatran (*n* = 13), we have followed over time miR-27a-3p expression and analyzed its levels at the time of diagnosis and prior of DOAC therapy (t0, baseline) and on the 7th day of anticoagulation (t1).
• DOAC treatment did not alter miR-27a-3p expression in the pooled population (0.80 fold-change, *p* = 0.486) or when patients were stratified per drug (0.839 fold-change, *p* = 0.706, for rivaroxaban; 0.921 fold-change, *p* = 0.800 for apixaban; 0.733 fold-change, *p* = 0.540 for dabigatran).
• Despite the regulatory role of miR-27a-3p on coagulation cascade, treatment with DOACs does not alter its expression.

## Introduction

Direct oral anticoagulants (DOACs) constitute the cornerstone of anticoagulation increasingly replacing the first oral anticoagulants, vitamin K antagonists (VKAs), in various conditions [1]. DOACs act directly on the coagulation cascade, overcoming, thus, the dosing challenges of VKAs including having a narrow therapeutic window and a wide interpatient variability in dosing requirements [1]. Despite the profound mechanism of DOACs on inhibiting coagulation pathway, less is known on the molecular mechanisms and the potential feedback loop homeostasis mechanism on the coagulation cascade provoked by DOAC mediated thrombin and factor Xa inhibition.

More recently, microRNAs (miRs), the small, non-coding RNAs that have been found to control post-transcriptionally gene expression, have emerged as attractive candidates to study the mechanisms of drug action and response to pharmacotherapy [2], leading to the introduction of the term “pharmacomiRNomics” [3]. The main characteristics of miRs that are taken into advantage in pharmacomiRNomics are their ability to regulate different pathways since they target multiple mRNAs, and their dynamic expression that is altered after exposure to endogenous and exogenous stimuli helping thus the cell to adapt in different situations such as in disease and drug treatment [4].

miRs have been extensively studied in cancer [5]. However, in anticoagulation, the role of miRs is not yet elucidated and the potential effect of miRs on DOAC treatment or the effect of DOACs on miR expression is just scarcely studied. Only two studies have been published in this field showing how baseline expression of miRs may affect pharmacokinetic and pharmacodynamic profile of DOACs. Specifically, it was shown that miR-320a-3p and miR-483-5p levels were associated with pharmacokinetic and pharmacodynamic profiles of rivaroxaban in healthy Chinese subjects [6], whereas miR-142 and miR-39 expression levels were not associated with rivaroxaban plasma concentration [7].

Evidence shows that miRs hold a role in hemophilia and in bleeding disorders beyond hemophilia, as well as in arterial and venous thrombosis by targeting mRNAs encoding coagulation factors [8] and that groups of miRs specifically target procoagulant, anticoagulant, as well as fibrinolytic components genes supporting a miRNA-mediated regulation of the hemostatic system [9]. Based on the online database for prediction of functional microRNA targets, miRDB [10], miR-27a is such a miR; it targets proteins on the coagulation cascade, including the tissue factor pathway inhibitor (TFPI), tissue factor pathway inhibitor 2 (TFPI2), and tissue factor (TF), regulating thus blood coagulation. TFPIs inhibit factor Xa (anticoagulant state), whereas TF triggers the initiation of thrombin formation (procoagulable state). Therefore, it can be assumed that the effect of DOACs, if any, on miR-27a expression differs between factor Xa inhibitors and dabigatran.

In the present study we have hypothesized that treatment with DOACs may alter the expression of miR-27a-3p leading to a broader regulation of the coagulation cascade. The primary aim of our study is to study whether there is any change of miR-27a-3p expression in patients with non-valvular atrial fibrillation (AF) treated with rivaroxaban, apixaban or dabigatran. Secondary aims of the study include (a) the analysis of differences between direct oral thrombin and factor Xa inhibitor type of DOACs on miR-27a-3p expression and (b) the potential effect of sex, bleeding, and of common comorbidities on miR-27a-3p expression.

## Patients & methods

### Study population

**Fig. 1:**
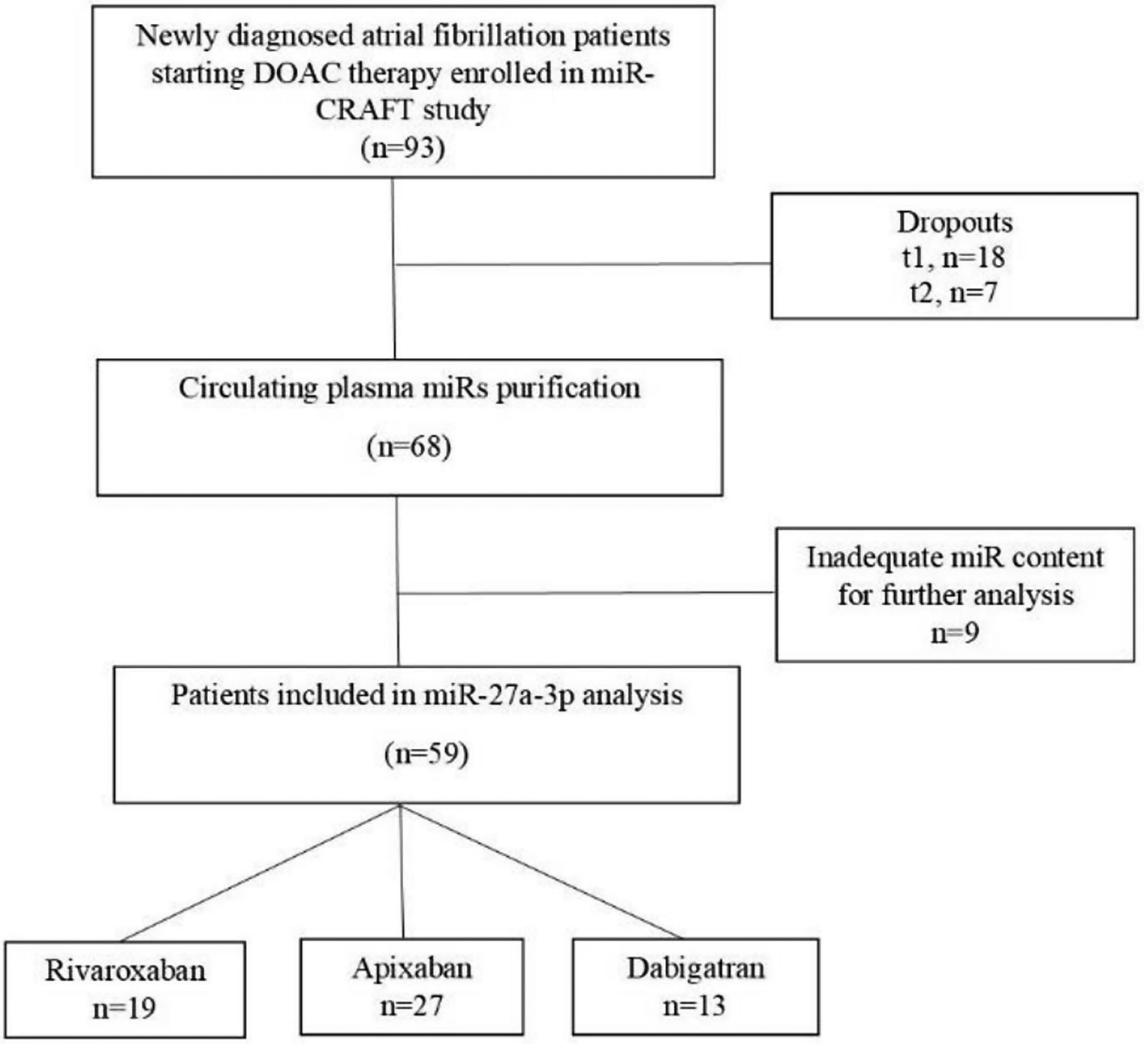
Analytic flowchart of miR-CRAFT patient subgroup included in miR-27a-3p expression analysis study

A total of 59 AF patients, a subgroup of participants of miR-CRAFT study, were included in miR-27a-3p expression analysis. An analytic flowchart of patients included in the study is presented in Fig. 1.

miR-CRAFT study design has been described earlier [11]. In brief, naïve AF patients initiating DOAC treatment with rivaroxaban, apixaban or dabigatran are prospectively enrolled and monitored at baseline and after seven and twenty-eight days of DOAC treatment. Patients age under 18 years old, cancer and insulin treatment are the exclusion criteria of the study. Adverse events, including thromboembolic and bleeding events (even minor, non-clinically significant bleedings), are recorded. All subjects participate after being informed about the study and giving written consent. The study protocol was approved by Ethical Committee of Athens General Hospital “Elpis” (approval ΕΣ 23/14 April 2019) and of the Academic General Hospital of Alexandroupolis (approval ΕΣ 3/3 February 2022).

### MiR purification and miR-27a-3p expression analysis

From all participants, blood samples (approximately 3 mL) were collected by direct venipuncture into a vacutainer tube containing ethylenediaminetetraacetic acid (EDTA) at the time of diagnosis (t0, baseline), and on the 7th day of anticoagulation (t1). Blood samples on t1 were drawn in the morning and prior to the scheduled drug dosage (trough levels). Fresh blood sample was centrifuged within 1 h of collection for 10 min at 2000× *g*; plasma was removed without disturbing sedimented cells and stored at − 80 °C. Circulating plasma miRs were isolated by use of NucleoSpin^®^ miRNA Plasma (Macherey-Nagel, Düren, Germany). miR quantity was assessed by Qubit 4 fluorometer (ThermoFisher Scientific) by use of QUBIT MICRORNA ASSAY KIT (ThermoFisher Scientific) and all samples were diluted to a final concentration of 0,5ng/µl. cDNA was synthesized for samples corresponding to t0 and t1 by use of TAQMAN ADV MICRORNA CDNA SYN (ThermoFisher Scientific) with an initial input of 1ng (2 µl) of each miR sample. Quantitative RT-PCR for miR-27a-3p was performed using a predesigned TaqMan assay (478384_miR, ThermoFisher Scientific). miR-16-5p (477860_miR, ThermoFisher Scientific) that is consistently expressed in plasma [12] was used as an endogenous control for data normalization. Each reaction was carried out in 96-well plates on QuantStudio™ 12 K Flex Real-Time PCR System (ThermoFisher Scientific).

Each reaction was carried out in 10 µL of a total reaction volume containing 5 µL of TaqMan™ Fast Advanced Master Mix, 0.5 µL of 20x TaqMan assay, 2,5 µl of 1:10 diluted cDNA template and 2 µl RNase-free water. qPCR conditions were the following: 95 ° C for 20 s (enzyme activation), and 40 cycles of 95 ° C for 1 s (denature) and 60 ° C for 20 s (anneal/extend). Non-template controls for both miR assays were included in each plate. All reactions were performed in duplicates.

QuantStudio 12 K Flex Software v1.5 was used to verify the amplification. Replicates with a Ct standard deviation greater than 1, or with an average Ct greater than 35, were omitted from further analysis.

Relative Quantitation (RQ) (2^−∆∆Ct^, where Ct = threshold cycle, delta Ct = Ct _miR−27a−3p_ minus Ct _mir−16−5p_, Delta Delta Ct = ∆Ct _interrogation group_– ∆Ct _reference group_) was calculated using ExpressionSuite Software Version 1.3 (Applied Biosystems).

### Statistical analysis

Shapiro–Wilk test was used to assess normality of continuous variables. Continuous variables are expressed as mean ± standard deviation (SD) in the case of normal distribution, otherwise they are expressed as median (25th, 75th percentiles). Based on normality, parametric (independent t-test or one-way ANOVA) and non-parametric (Mann–Whitney test or Kruskal–Wallis test) tests were used to compare continuous variables between two or more groups, as appropriate using the IBM SPSS Statistics for Windows, Version 27.0 (Armonk, NY: IBM Corp.). Subgroups were created and miR-27a-3p expression was compared within different DOACs, sex, bleedings and comorbidities using 2^−∆∆Ct^ method. A p-value less than 0.05 was considered statistical significant.

## Results

### Patient characteristics

Patient demographic, biochemical and clinical characteristics are presented in Table 1. The cohort population consists of 59 AF patients (55.9% male) of mean age 70 years (± 12). Median CHA_2_DS_2_-Vasc Score was 3, whereas 30 patients (50.8%) had impaired renal function (10% of whom with renal failure). Patients were treated with rivaroxaban (*n* = 19), apixaban (*n* = 27) or dabigatran (*n* = 13). No major bleeding or thrombotic events were recorded. A total of 14 minor bleeding events occurred. Among drug and bleeding categories, no differences were noticed in patient characteristics (data not shown).

**Table 1:** Demographic and clinical characteristics of patient population (*n* = 59)

Characteristics	Values
**Demographic**	
Male (n, %)	33 (55.9)
Age (years, mean ± SD)	70 ± 12
Weight (kg, median, 25, 75 percentiles)	78 (62, 87)
Height (cm, median, 25, 75 percentiles)	168 (155, 175)
Smokers (n, %)	12 (20.3)
**Biochemical**	
Hemoglobin (gr%, median, 25, 75 percentiles)	13.85 (12.42, 15.12)
Platelets (100/µl, mean ± SD)	230.9 ± 69.1
Urea (mg/dl, median, 25, 75 percentiles)	40 (33, 57)
Creatinine (mg/dl, median, 25, 75 percentiles)	0.95 (0.79, 1.19)
Creatinine Clearance (ml/min, median, 25, 75 percentiles)	72.8 (49.0, 97.9)
SGOT (U/L, median, 25, 75 percentiles)	21 (18, 30)
SGPT (U/L, median, 25, 75 percentiles)	25 (20, 35.75)
**Clinical**	
CHA_2_DS_2_-Vasc Score (median, 25, 75 percentiles)	3 (2, 4)
Renal function	
Normal (n, %)	22 (42.3)
Impaired (n, %)	27 (51.9)
Failure (n, %)	3 (5.1)
**DOAC therapy**	
Rivaroxaban (n, %)	19 (32.2)
Rivaroxaban 15/20 mg (n, %)	5 (26.3) /14 (73.7)
Apixaban (n, %)	27 (45.8)
Apixaban 2.5/5 mg (n, %)	9 (33.3) / 18 (66.7)
Dabigatran (n, %)	13 (22.0)
Dabigatran 110/150 mg (n, %)	2 (15.4) / 11 (84.6)
Bleeding (n, %)	14 (23.7)
**Co-morbidities**	
Hypertension (n, %)	36 (61.0)
Type 2 Diabetes (n, %)	16 (27.1)
Dyslipidemia (n, %)	31 (52.5)
History of stroke (n, %)	3 (5.0)

SD; standard deviation

### miR-27a-3p expression after 7 days of DOAC treatment

**Fig. 2:**
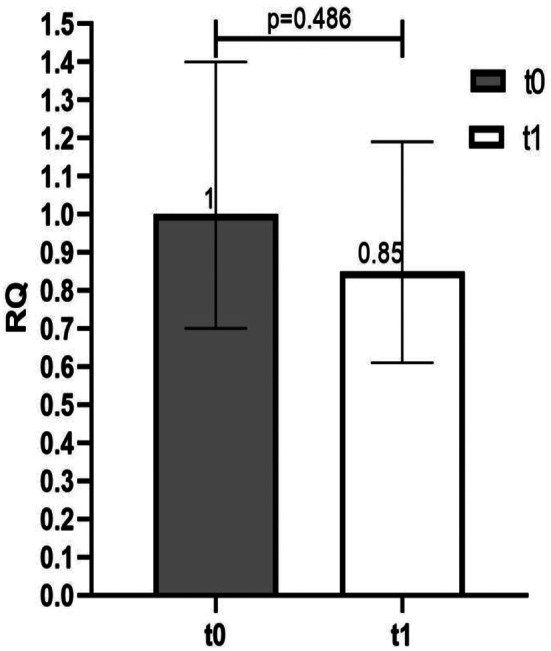
Change of miR-27a-3p expression after 7 days of DOAC therapy. miR-27a-3p expression in DOAC treated patients at baseline (t0) and after 7 days of DOAC treatment (t1). Error bars represent the range of relative quantitation (RQ) values. RQ, relative quantitation

In the total population, miR-27a-3p expression was not altered from baseline after 7 days of DOAC treatment (*p* = 0.486), albeit a 0.15 fold-decrease in its expression was noticed (Fig. 2).

**Fig. 3:**
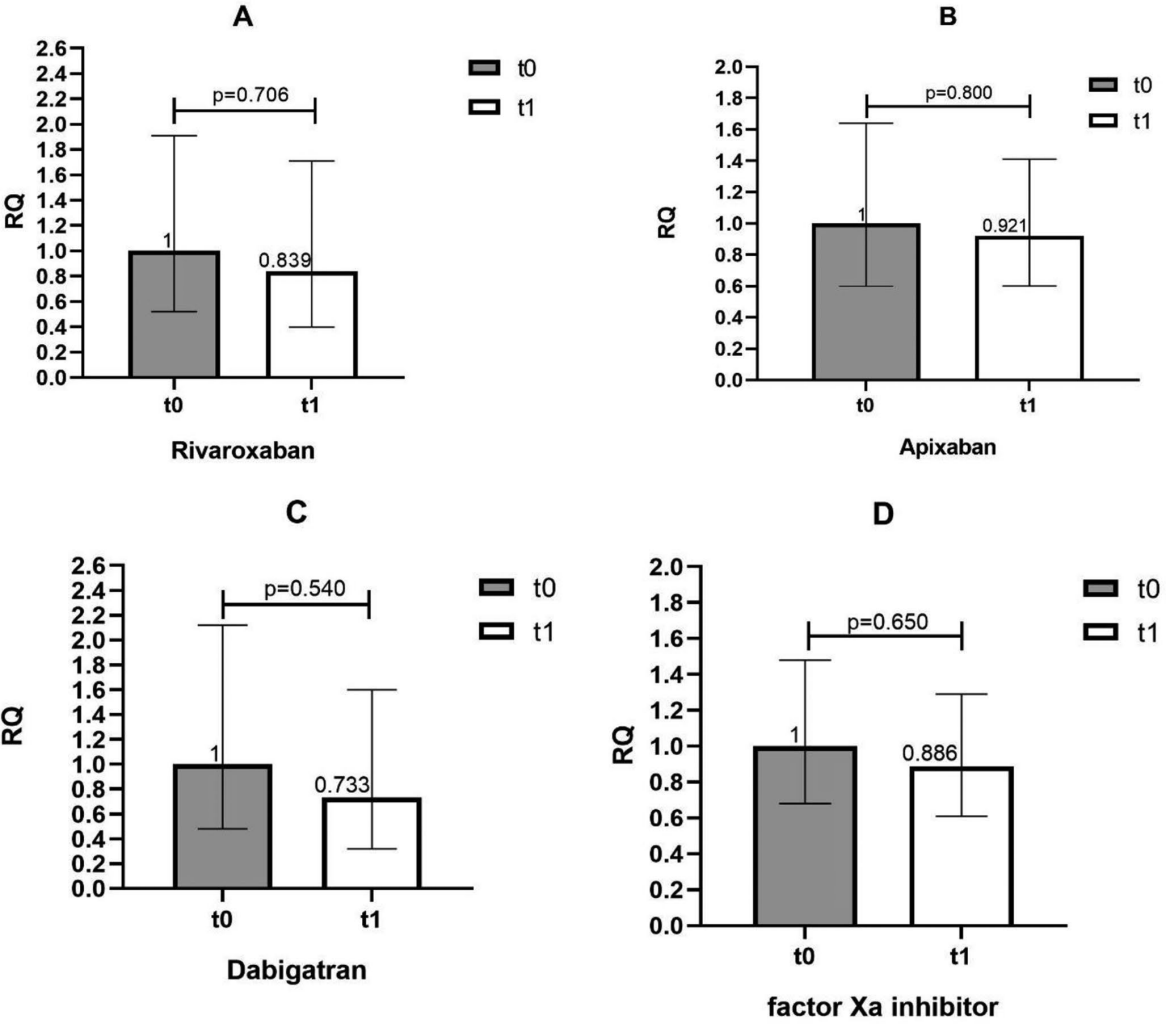
Change of miR-27a-3p expression after 7 days of DOAC therapy, stratified per treatment (**A**, rivaroxaban; **B**, apixaban; **C**, dabigatran; **D**, factor Xa inhibitors). miR-27a-3p expression at baseline (t0) and after 7 days of DOAC treatment (t1) in rivaroxaban (**A**), apixaban (**B**), dabigatran (**C**) and factor Xa inhibitor (**D**) treated patients. Error bars represent the range of relative quantitation (RQ) values. RQ, relative quantitation.

Patients were stratified according to each drug of category and, additionally, patients treated with factor Xa inhibitors were grouped together. miR-27a-3p expression did not differ on 7 days of therapy in any treatment category (Fig. 3).

### miR-27a-3p expression between bleeding cases and controls

**Fig. 4:**
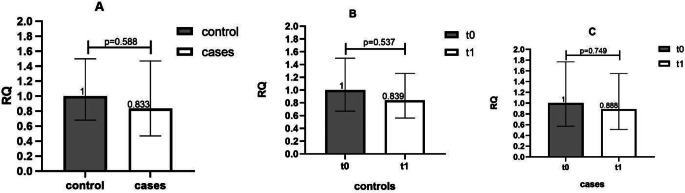
Comparison of miR-27a-3p expression between controls and bleeding cases at baseline (t0) (**A**) and in non-bleeding controls (**B**) and in bleeding cases (**C**) at baseline (t0) and after 7 days of DOAC treatment (t1). miR-27a-3p expression at baseline in DOAC treated patients experiencing (cases) or not (controls) bleeding complications (**A**) and after 7 days of DOAC therapy in controls (**B**) and bleeding cases (**C**). Error bars represent the range of relative quantitation (RQ) values. RQ, relative quantitation.

To assess whether the expression of miR-27a-3p predisposes AF patients to bleedings or whether bleeding per se mechanistically alters its expression, patients were further stratified into bleeding cases (*n* = 14) and controls (*n* = 45). Baseline expression of miR-27a-3p did not differ between cases and controls (Fig. 4A). Additionally, bleeding was not associated with altered miR-27a-3p expression both in pooled DOAC-treated population (Fig. 4B and C) as well as per drug of the category (factor Xa inhibitors vs. dabigatran) (data not shown).

### Sex effect on miR-27a-3p expression

In sex analysis, at baseline (t0) we have observed that female patients had a 1.564 fold-increased miR-27a-3p expression compared to male patients, however, this difference did not reach statistical significance (*p* = 0.177).

Seven days of DOAC therapy did not alter miR-27a-3p expression in male patients (1.023 fold-change, *p* = 0.946), whereas in female patients a trend towards reduced expression was noticed (0.683 fold-change, *p* = 0.243). When patients were stratified per sex and DOAC mechanism of action, in female factor Xa inhibitor treated patients (*n* = 16) miR-27a-3p expression was decreased after 7 days of treatment (0.594 fold-change, *p* = 0.149), whereas in male patients miR-27a-3p expression was not altered (1.097 fold-change, *p* = 0.795). Due to the low sample size after sex stratification, expression analyses were not performed for dabigatran (9 male patients and 4 female patients) as well as for each factor Xa inhibitor.

### Comorbidities effect on baseline miR-27a-3p expression

To further assess whether comorbidities alter miR-27a-3p expression at baseline and, thus, interfere or mask the effect of DOACs on its expression, we have stratified patients according to having or not hypertension, type 2 diabetes and dyslipidemia. In every case, no difference in miR-27a-3p expression was present (hypertension vs. controls 1.278 fold-change, *p* = 0.477; type 2 diabetes vs. controls 0.843 fold-change, *p* = 0.666; dyslipidemia vs. controls 0.946 fold-change, *p* = 0.849).

## Discussion

In the present study we have analyzed the potential effect of DOAC treatment on miR-27a-3p expression in naïve AF patients, a subgroup of miR-CRAFT study participants, starting oral anticoagulation with rivaroxaban, apixaban or dabigatran. To the best of our knowledge, this is the seminal study to study miR-27a-3p expression over time in DOAC treated AF patients. Our results show that 7 days of DOAC therapy did not alter miR-27a-3p expression either in pooled population or after stratifying patients by DOAC, sex, bleedings and comorbidities.

miR-27a, generating the mature miR-27a-3p and miR-27a-5p microRNAs, has been characterized as an oncogenic miR [13] and, more recently, as a contributing factor to toxicity incidence to fluoropyrimidines via inhibition of dihydropyrimidine dehydrogenase encoding gene (DPYD) [14]. miR-27a-3p is the major isoform targeting nearly 1,500 genes versus 251 targets of miR-27a-5p isoform [10]. Beyond its role in cancer, miR-27a-3p targets span different molecular pathways, including the coagulation cascade. More specifically, among the proteins implicated in blood coagulation pathway, miR-27a-3p regulates the expression of TFPI (miRDBD target score 72), TF (miRDBD target score 57) and TFPI2 (miRDBD target score 56) [10]. TFPIs directly inhibit factor Xa, the target of rivaroxaban and apixaban, and TF-FVIIa, preventing thus the formation of an active prothrombinase and regulating the generation of thrombin [15, 16]. Additionally, TFPIs modulate the severity of a wide variety of bleeding and clotting disorders [17]. TF, the initiator of blood coagulation, triggers the initiation of thrombin formation [18]. Given the crucial role of both TFPIs and TF on coagulation homeostasis maintenance, the rational of our hypothesis in the present study is that the DOAC inhibiting effect on factor Xa and thrombin results to an amplification reaction towards anticoagulation that mechanistically involves the altered expression of miR-27a-3p and the modulation, thus, of TFPIs and TF expression.

The results of our study show that 7 days of DOAC treatment do not alter the expression of miR-27a-3p either in the pooled patient population or per drug of DOAC drug class. Since this is the seminal study on the potential effect of DOACs on mir-27a-3p expression, no further comparisons can be made with the published literature. In the field of AF, however, the potential use of miRs as circulating biomarkers of the disease increasingly gains attention [19]. miR-27a-3p was highlighted as a co-DEG AF-related stroke in a bioinformatic gene analysis seeking for potential biomarkers and therapeutic targets of this condition [20]. More recently, in a study aiming to investigate the regulatory effect of lncRNA GAS5 on the electrical remodeling of neonatal rat cardiomyocytes induced by rapid pacing, Xi et al. have shown that the overexpression of GAS5 attenuated electrical remodeling and that miR-27a-3p had a direct negative regulatory effect on lncRNA-GAS5 [21]. Though this is still a naïve field, the potential role of miR-27a-3p on AF incidence merits further investigation. Additionally, the heterogeneity between various DOACs on their pleiotropy [1], prompted us to assess whether there is a different effect on miR-27a-3p expression between thrombin inhibitor and factor Xa inhibitors, as well as among different types of factor Xa inhibitors. Our results do not provide evidence for such an effect, however, results should be replicated in larger studies.

Recent evidence suggests that sex-differences exist in AF pathophysiology [22]. Additionally, a sex-specific expression of miRs has been recognized in both physiological and pathological processes [23]. For miR-27a-3p, sex-specific correlations that were dependent on cancer stage were observed in female patients [24]. To reduce sex-confounding in our study, we have compared the baseline miR-27a-3p expression between male and female AF patients. We have found that female patients presented at baseline with higher miR-27a-3p expression compared to male patients and a reduction of its expression after 7 days of DOAC treatment was found only in female patients, however none of these differences reached statistical significance. To further study the potential effect of sex on miR-27a-3p expression, larger studies are necessary.

Similarly to sex, and according to published literature, it cannot be excluded that other common comorbidities may interfere with miR-27a-3p expression. Some evidence exists linking the expression of miR-27a with hypertension [25, 26], type 2 diabetes [27, 28] and dyslipidemia [29, 30]. In our study, we did not find a different in miR-27a-3p expression in either situation, suggesting that hypertension, type 2 diabetes and dyslipidemia are not confounding factors in the study population.

Our study has several strengths. miR-CRAFT is a well-controlled study, and the study population is balanced for two major confounders of miR expression, cancer and insulin treatment. For cancer, it is well known that the expression of several miRs can be up- or down-regulated and oncology is currently the field of medicine that benefits the most from the characterization of miRs that can be used as prognostic markers of disease. Increasing evidence shows that miR-27a plays a vital role in tumor biology acting either as an oncogene or a tumor suppressor gene depending on the type of cancer [31] and that miR-27a-3p is abnormally expressed in various types of cancers [32]. Recently, it has been shown that insulin treatment induced the downregulation of miR-27a-3p expression in human granulosa-like tumor cell line [33]. These confounders are both patient exclusion criteria to reduce confounding [11]. Moreover, both the intraday and intraindividual day-to-day variances of miR expression are currently scarcely studied. For selected miRs, such as miR-125a, miR-146a, miR-155, let-7e and miR-106a evidence proposes that no common circadian variances exist in each miR expression in plasma, however, intraindividual day-to-day variances in miR expression in plasma were noticed [34]. In our study, in order to reduce the possibility of such variability, we have used strict criteria for sampling timing, and all blood samples on 7 days of DOAC treatment were drawn in the morning and prior to the scheduled drug dosage [11]. Additionally, in our study other comorbidities did not affect miR-27a-3p expression. miR-CRAFT study is adequately powered to detect a 1.5 fold-change in miR expression with an 80% average power within groups.

However, it should be acknowledged, that several limitations also exist. Mainly, the results should be interpreted with caution, especially in sub-group analyses due to low sample size, to avoid false negative results. The design of the study is focused on the effect of DOACs on miR expression and, thus, other endpoints such as the role of miR-27a-3p in AF-related coagulopathy and thrombosis risk cannot be assessed. We herein report results of the effect of DOAC treatment on miR-27a-3p expression at 7 days of therapy. Since miRs respond quickly to stimuli such as drug treatment, we did not further seek for differences at 28 days. However, the lack of association in early response found in our study cannot exclude a potential late response effect of DOAC treatment on miR-27a-3p. Moreover, despite the finding that DOACs do not alter miR-27a-3p expression, TF and TFPI could be both altered by DOAC treatment irrespectively of miR-27a-3p regulation pathway; however, this analysis is beyond the scopes of our study.

## Conclusions

In conclusion, our study provides seminal insights on the effect of DOAC therapy on miR-27a-3p expression. The results show that despite the regulatory role of miR-27a-3p on coagulation cascade, treatment with DOACs does not alter its expression. However, additional studies in different ethnic groups are necessary to fully elucidate the effect, if any, of DOACs on miR-27a-3p expression.

## Data Availability

The original contributions presented in this study are included in the article. Further inquiries can be directed to the corresponding author.
